# Ion-Selective Scattering Studied Using the Variable-Energy Electron Irradiation in the Ba_0.2_K_0.8_Fe_2_As_2_ Superconductor

**DOI:** 10.3390/ma16134520

**Published:** 2023-06-22

**Authors:** Kyuil Cho, Marcin Kończykowski, Makariy A. Tanatar, Igor I. Mazin, Yong Liu, Thomas A. Lograsso, Ruslan Prozorov

**Affiliations:** 1 Ames National Laboratory, Ames, IA 50011, USA; 2 Department of Physics, Hope College, Holland, MI 49423, USA; 3 Laboratoire des Solides Irradiés, CEA/DRF/IRAMIS, École Polytechnique, CNRS, Institut Polytechnique de Paris, F-91128 Palaiseau, France; 4 Department of Physics & Astronomy, Iowa State University, Ames, IA 50011, USA; 5 Department of Physics & Astronomy and Quantum Science & Engineering Center, George Mason University, Fairfax, VA 22030, USA; 6 Crystal Growth Facility, Institute of Physics, École Polytechnique Fédérale de Lausanne, CH-1015 Lausanne, Switzerland

**Keywords:** superconductivity, iron-based superconductor, irradiation, scattering, disorder, defect

## Abstract

Low-temperature variable-energy electron irradiation was used to induce non-magnetic disorder in a single crystal of a hole-doped iron-based superconductor, Ba${}_{1-x}$K${}_{x}$Fe${}_{2}$As${}_{2}$, *x* = 0.80. To avoid systematic errors, the beam energy was adjusted non-consequently for five values between 1.0 and 2.5 MeV when sample resistance was measured in situ at 22 K. For all energies, the resistivity raises linearly with the irradiation fluence suggesting the creation of uncorrelated dilute point-like disorder (confirmed by simulations). The rate of the resistivity increase peaks at energies below 1.5 MeV. Comparison with calculated partial cross-sections points to the predominant creation of defects in the iron sublattice. Simultaneously, superconducting $T_{c}$, measured separately between the irradiation runs, is monotonically suppressed as expected, since it depends on the total scattering rate, hence on the total cross-section, which is a monotonically increasing function of the energy. Our work experimentally confirms an often-made assumption of the dominant role of the iron sub-lattice in iron-based superconductors.

## 1. Introduction

Response of superconductivity to impurities and defects provides a useful tool to study the pairing mechanism of superconductors [1,2,3]. The isotropic *s*—wave paring state of conventional Bardeen–Cooper–Schrieffer (BCS) superconductors is robust against non-magnetic scattering. This statement is known as Anderson theorem [4]. However, in the case of paramagnetic impurities, scattering involves simultaneous flipping of the spins of impurity and conduction electron, destroying singlet Cooper pairs. Thus, according to the Abrikosov and Gor’kov theory [5], conventional BCS superconductivity is suppressed and is destroyed at the finite critical value of the magnetic dimensionless scattering rate, $\Gamma =\hbar /\left(2\pi k_{B}T_{c0}\tau \right)\approx 0.14$. In the cases of the anisotropic or multiband superconducting order parameters, even nonmagnetic scattering is pair-breaking and leads to a suppression of $T_{c}$ [6,7].

Traditionally, chemical doping and alloying are used to induce extra scattering [8]. However, in addition to changing the scattering rate, these cause changes in the electronic band structure and the Fermi energy level, and build internal “chemical pressure”, all of which affect the measurable properties.

Particle irradiation is an alternative way to generate scattering centers, and it has been intensively used to investigate the properties of materials. Depending on the choice of particles, the character of induced scattering centers varies from point-like defects, mostly vacancies (electron irradiation) [9,10,11,12,13,14,15,16,17], to dendritic clusters (proton irradiation) [18,19,20,21,22,23,24,25,26,27,28], and to columnar defects (heavy-ion irradiation) [29,30,31,32,33,34,35,36,37,38]. Furthermore, if the energy of the projectile particles varies, the character of defects generated also changes accordingly since the scattering dynamics significantly vary with the energy [39,40].

In this contribution, we use variable-energy electron irradiation to experimentally determine which ions contribute most to the scattering rate in iron-based superconductors, thus testing the models of electronic conductance in these materials. We chose Ba${}_{1-x}$K${}_{x}$Fe${}_{2}$As${}_{2}$ as one of the most intensively studied among the iron-based superconductors [16,41,42]. Here, superconductivity exists starting from x= 0.16 and extends all the way to x= 1. The abrupt change in the superconducting gap structure around x= 0.7 was attributed to the Lifshitz transition [13,43,44]. At low *x*, superconductivity coexists with long range magnetic order [45]. To avoid the influence of the magnetic phase and enable in situ resistivity measurements, performed at a fixed 22 K in our setup, we chose the overdoped compound with x= 0.8 with a convenient $T_{c,onset}=20.2$ K.

## 2. Materials and Methods

Single crystals of Ba${}_{0.2}$K${}_{0.8}$Fe${}_{2}$As${}_{2}$ were grown by using an inverted temperate gradient method with the starting materials—Ba and K lumps, and Fe and As powders. Details of the growth method can be found elsewhere [13,46]. Resistivity measurements were performed in a standard four-probe configuration. Typical dimensions of the samples are (1–2) × 0.5 × (0.02–0.1) mm${}^{3}$. Silver wires of 50 μm diameter were soldered to the sample to provide electrical contacts [47]. The sample was mounted on a Kyocera chip over a hole of about 5 mm diameter at the center. The Kyocera chip was transferred to the irradiation chamber filled with liquid hydrogen providing efficient cooling down to 22 K. A Faraday cup placed behind the chamber enabled accurate measurement of the fluence during irradiation. The electron irradiation was performed at the SIRIUS Pelletron facility of the Laboratoire des Solides Irradiés at the École Polytechnique in Palaiseau, France. The energy of the electron beam was varied from 1.0 MeV to 2.5 MeV. The acquired irradiation dose is conveniently measured in C/cm${}^{2}$, where 1 C/cm${}^{2}$ = 6.24 × 10${}^{18}$ electrons/cm${}^{2}$. After irradiation, the sample in the Kyocera chip was transferred to another set-up for temperature-dependent resistivity measurement.

## 3. Results and Discussion

**Figure 1:**
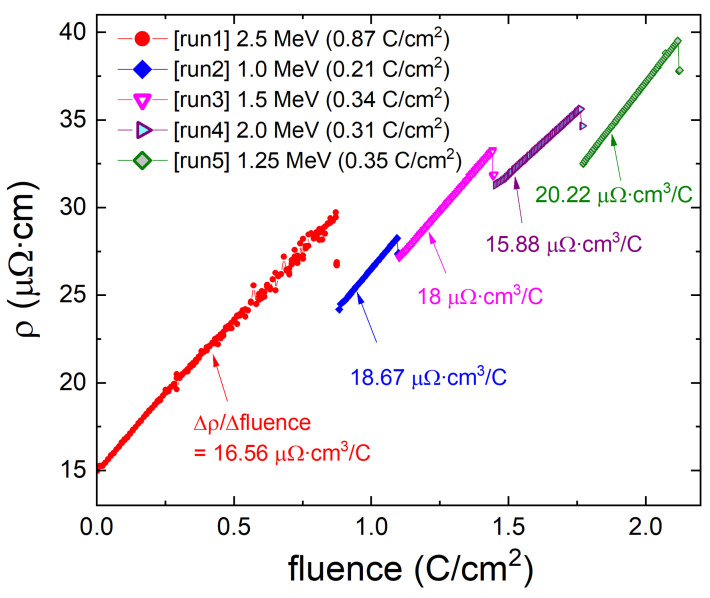
Fluence dependence of the resistivity of a Ba${}_{0.2}$K${}_{0.8}$Fe${}_{2}$As${}_{2}$ single crystal measured in situ in an irradiation chamber during electron irradiation. The sample was sitting in a liquid hydrogen environment at a temperature around *T* = 22 K. Five irradiation runs were conducted in order (sections of the broken line in the figure). After each irradiation, the sample was taken out of the irradiation chamber for characterization and returned for the next irradiation. The sample’s thermal cycling to room temperature resulted in a partial disorder annealing and a slight resistivity decrease compared to the value at the end of the previous run.

Figure 1 shows the in situ resistivity measurement during irradiation. The electron irradiation was performed at T= 22 K in liquid hydrogen. A low temperature is needed to remove the heat generated during irradiation, prevent immediate recombination of Frenkel pairs and, importantly, prevent clusterization and agglomeration of the produced defects. The first irradiation (run 1) with a 2.5 MeV electron beam was conducted up to 0.87 C/cm${}^{2}$. During this irradiation, the resistivity monotonically increased from 15 to 30 μΩcm. The rate of resistivity increase per fluence (Δρ/Δfluence) was 16.56 μΩcm${}^{3}/C$. After run 1, the sample was removed from the irradiation chamber and transferred to the other cryostat to measure the temperature-dependent resistivity. For the second irradiation (run 2), the sample was again mounted to the irradiation chamber. Between run1 and run 2, the sample was exposed to the room temperature and annealing of defects at room-temperature was evident as a decrease in resistivity from 30 to 24 μΩcm. Run 2 was performed with a 1.0 MeV electron beam up to 0.21 C/cm${}^{2}$. The identical procedure was repeated for all five irradiation runs in order.

**Figure 2:**
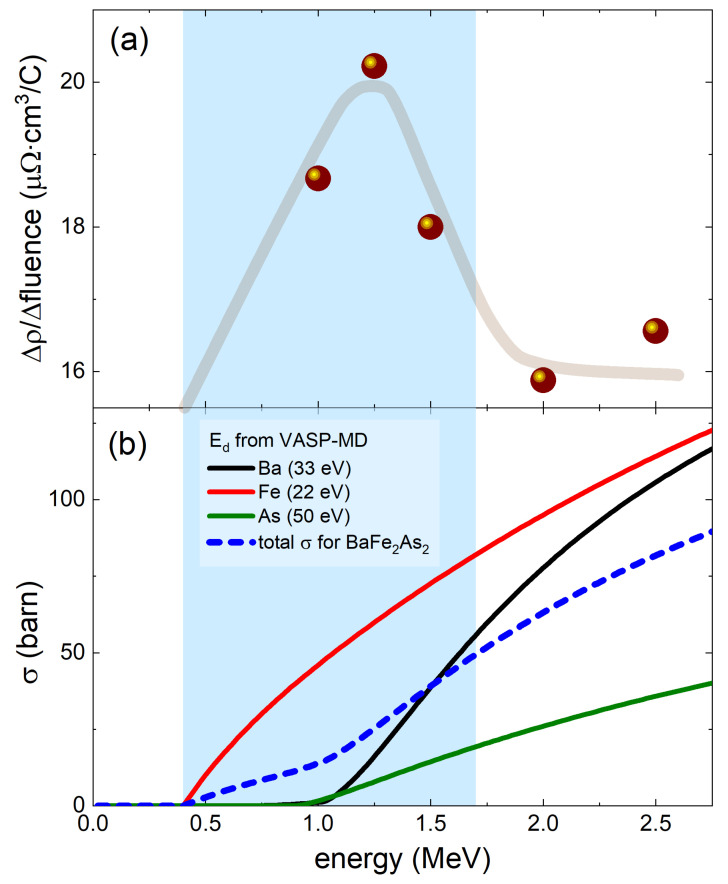
(**a**) Rate of the in situ resistivity increase with the fluence (slope of the line’s segments in Figure 1) plotted as a function of the energy of the electron beam. Δρ is the increase in resistivity during each irradiation run and Δfluence is the total fluence for that irradiation. The lower energy irradiations show a larger rate of in situ resistivity per fluence. (**b**) Energy-dependent scattering cross-sections for Ba, Fe, and As are calculated using the displacement energies (E${}_{d}$) = 33 eV (Ba), 22 eV (Fe), and 50 eV (As), which were calculated using VASP-MD simulation. The total cross-section for BaFe${}_{2}$As${}_{2}$ is plotted as a dashed line.

Figure 2a summarizes the energy dependence of the in situ resistivity found in Figure 1. Interestingly, we found that the rate of change in in situ resistivity, Δρ/Δfluence, is substantially larger for the irradiation at lower energies. To understand this behavior, we need to calculate the energy-dependent partial cross-section for Ba, Fe, and As. This requires knowledge of the knockout barriers, $E_{d}$, which depend on the element and on its position in a particular crystal lattice. The knockout threshold barriers’ $E_{d}$ values, Ba (33 eV), Fe (22 eV), and As (50 eV), were estimated by using projector-augmented wave [48] as implemented in the Vienna Ab-initio Simulation Package (VASP) [49]. Gradient correction [50] was used in the calculations, and semicore Ba-s and Fe-p states were treated as valence states. We used a supercell of 18 formula units and 1 K-point in the Brillouin zone. Ab initio molecular dynamics (MD) was performed using the standard VASP settings [51]. Calculations were initialized by assigning a prescribed kinetic energy to a given atom and monitoring whether it will drift away in the process of MD, or return back to its original site. The magnetic state of the starting configuration did not affect the final estimate of the knockout energy within the accuracy that we were interested in. With the obtained $E_{d}$ values, we used SECTE (“Sections Efficaces Calcul Transport d’Électrons”) software, developed at École Polytechnique (Palaiseau, France) by members of the “Laboratoire des Solides Irradiés”, specifically for the interpretation of MeV-range electron irradiation. Essentially, this is a computer-assisted atomic-weights-averaged interpolation of the ion knockout cross-sections tabulated by O. S. Oen [52]. It appears that the defects produced roughly below 1.5 MeV contribute most to the resistivity change and, according to our calculations, these are defects in the iron sublattice. This is our central profound result, which has always been assumed in iron-based superconductors, but is now directly experimentally verified.

**Figure 3:**
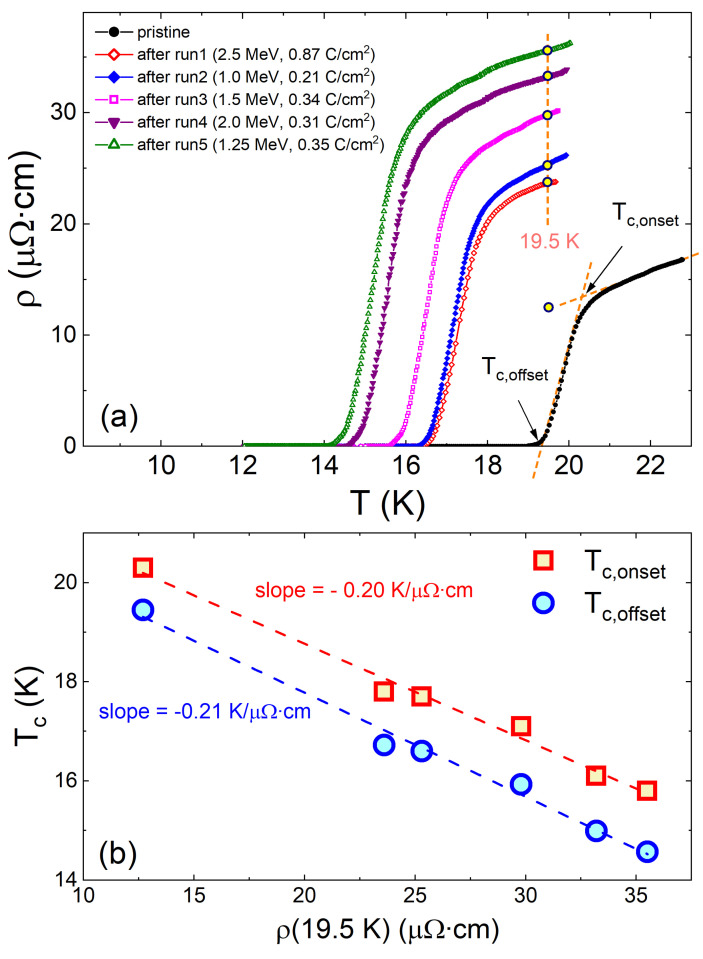
(**a**) Temperature-dependent resistivity measured after each run of irradiation. The sample was removed from the irradiation chamber and transferred to a separate set-up to measure the temperature-dependent resistivity. The normal state resistivity at 19.5 K is used as a parameter to indicate the amount of impurities generated upon irradiation. The definitions of $T_{c,onset}$ and $T_{c,offset}$ are shown as red dotted lines. (**b**) $T_{c}$ versus resistivity at *T* = 19.5 K. $T_{c}$ decreases at a rate of −0.20 K/μΩcm ($T_{c,onset}$) and −0.21 K/μΩcm ($T_{c,offset}$).

As a next step, we look at the independent parameter that depends on disorder—the superconducting transition temperature, $T_{c}$. Figure 3a shows the temperature-dependent resistivity measurement after each irradiation run. The first measurement (pristine) was conducted before irradiation. It has $T_{c,onset}$ = 20.2 K and $T_{c,offset}$ = 19.3 K. After each irradiation, the normal state resistivity increased, indicating the addition of defects. We used the normal state resistivity at 19.5 K, just above the transition, to characterize impurity scattering. Since the $T_{c}$ of the pristine samples is higher than 19.5 K, we used an extrapolation of the normal state resistivity down to 19.5 K to estimate the normal state resistivity. Figure 3b shows the suppression of $T_{c,onset}$ and $T_{c,offset}$ plotted against the normal state resistivity at *T* = 19.5 K. In general, $T_{c}$ decreases at a rate of −0.20 K/μΩcm ($T_{c,onset}$) and −0.21 K/μΩcm ($T_{c,offset}$). As expected, $T_{c}$ is affected by the total increase in resistivity, i.e., the total scattering rate. Defects in all ion sub-lattices contribute to scattering and therefore we should expect that the rate of $T_{c}$ suppression depends on the total cross-section.

**Figure 4:**
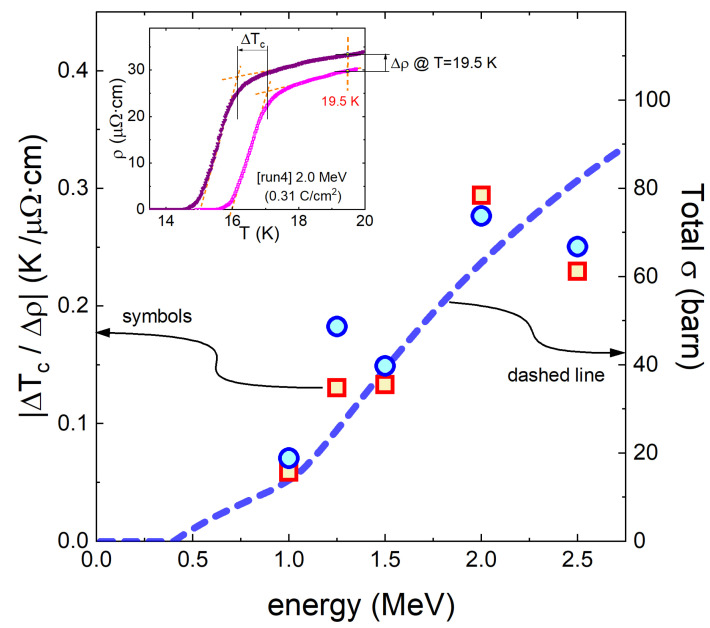
Normalized $T_{c}$ suppression rate ($|\Delta T_{c}/\Delta \rho |$) as a function of energy, calculated from the data in Figure 3a. The inset shows the definition of $\Delta T_{c}$ and Δρ for a particular run, ‘run4’ (2 MeV irradiation, 0.31 C/cm${}^{2}$), as an example. The fact that the suppression rate increases for higher energies is consistent with the increasing total cross-section (dashed line).

The $T_{c}$ suppression is further analyzed in Figure 4. The inset of Figure 4 explains the way the normalized suppression was calculated during the fourth irradiation with 2.0 MeV and 0.31 C/cm${}^{2}$ (‘run4’) as an example. $\Delta T_{c}$ is the variation in $T_{c}$ before and after 2.0 MeV irradiation, and Δρ is the variation in the resistivity measured at *T* = 19.5 K before and after 2.0 MeV irradiation. From these values, we calculated a normalized $T_{c}$ suppression rate of $|\Delta T_{c}/\Delta \rho |$. The same calculation was performed for all five irradiations and the results are plotted in the main panel of Figure 4. Indeed, the normalized $T_{c}$ suppression rate increases with increasing energy. As asserted above, this is expected since the total cross-section (dashed line) increases with energy.

## 4. Conclusions

Low-temperature variable-energy electron irradiation was used to probe ion-specific scattering and superconductivity in a single crystal of Ba${}_{1-x}$K${}_{x}$Fe${}_{2}$As${}_{2}$, *x* = 0.80. Measured in situ at 22 K, the rate of the resistivity increase peaks at electrons energies below 1.5 MeV. The comparison with the calculated partial cross-sections points to the predominant creation of defects in the iron sublattice at these energies. Simultaneously, superconducting $T_{c}$, measured separately between the irradiation runs, is monotonically suppressed with resistivity increase. This observation reflects that the total scattering rate on all defects, hence the total cross-section, monotonically increases with energy. Our work experimentally confirms an often-made assumption of the dominant role of the iron sub-lattice in scattering in iron-based superconductors.

## Data Availability

The data presented in this study are available on reasonable request from the corresponding author.
